# Supplementary material for: RNA language models predict mutations that improve RNA function
Source: bioRxiv. 2024 Apr 6:2024.04.05.588317. Preprint. [Version 1] doi: 10.1101/2024.04.05.588317 (PMC11014562; doi:10.1101/2024.04.05.588317)
Supplement: Supplementary file 2 [file media-6.pdf]

Supplementary Table 6. DNA primers used in this study

Primer Name	Sequence	Comment
U2473C-U2474C_F	CAAGAGCCCATATCGACGGCGGTGTTTGG	Forward primer for U2473C-U2474C 23S rRNA mutagenesis
U2473C-U2474C_R	CGATATGGGCTCTTGGGCGGTATCAGCCTG	Reverse primer for U2473C-U2474C 23S rRNA mutagenesis
U2477C_F	CAAGAGTTCACATCGACGGCGGTGTTTGG	Forward primer for U2477C 23S rRNA mutagenesis
U2477C_R	CGATGTGAACTCTTGGGCGGTATCAGCC	Reverse primer for U2477C 23S rRNA mutagenesis
H92-CC_F	ATGGCTGCCCGCCATTTAAAGTGGTACG	Forward primer for U2554C-U2555C 23S rRNA mutagenesis
H92-CC_R	ATGGCGGGCAGCCATACCCTTGGG	Reverse primer for U2554C-U2555C 23S rRNA mutagenesis
MS2_quant_F	CTTGCCCCGAGATGAGTTCTCCC	RT-PCR primer for the ribosome purity assay
MS2_quant_R	GTACCGGTTAGCTCAACGCATCGCT	RT-PCR primer for the ribosome purity assay
